# Anthelmintic medication with focus on first-trimester exposure: an evaluation of pregnancy outcomes based on the Embryotox cohort

**DOI:** 10.1038/s41598-026-57350-3

**Published:** 2026-06-14

**Authors:** Laura Heineke, Evelin Beck, Reinhold Kreutz, Katarina Dathe

**Affiliations:** 1 Charité – University Medical Center Berlin, corporate member of Freie Universität Berlin and Humboldt-Universität zu Berlin, Institute of Clinical Pharmacology and Toxicology, Embryotox Center of Clinical Teratology and Drug Safety in Pregnancy, Augustenburger Platz 1, 13353 Berlin, Germany; 2 Charité – University Medical Center Berlin, corporate member of Freie Universität Berlin and Humboldt-Universität zu Berlin, Institute of Clinical Pharmacology and Toxicology, Charitéplatz 1, 10117 Berlin, Germany

**Keywords:** Anthelmintics [Mesh], Pregnancy outcome [MeSH], Congenital abnormalities [MeSH], Risk assessment [Mesh], Diseases, Health care, Medical research, Risk factors

## Abstract

The present study aims to contribute to the available experience on the systemic use of five different anthelmintics during pregnancy (albendazole, mebendazole, praziquantel, pyrantel and pyrvinium). Particular focus was placed on the occurrence of major birth defects and pregnancy loss following first-trimester exposure. As the assessed agents differ considerably in terms of their chemical structure and mechanism of action, they were analyzed separately. A total of 282 pregnancies with exposure to the study medication were recorded in the Embryotox database (January 1, 2000**–**February 28, 2023) and analyzed descriptively. Among 75 live-born infants with first-trimester exposure to mebendazole, five major malformations were reported, three of which were heart defects. Of the 21 live-born infants exposed to pyrantel in the first trimester, two were affected by aplasia cutis congenita of the scalp, one of whom was co-exposed to thiamazole. Among 47 live-born children with first-trimester pyrvinium exposure, one major birth defect was reported. No major malformations were observed among 4 and 10 live-born children exposed to praziquantel and albendazole, respectively. Given the limitations, the findings should be interpreted as descriptive observations and possible signals only. Further investigations with larger cohorts are required.

**Supplementary Information:**

The online version contains supplementary material available at 10.1038/s41598-026-57350-3.

## Introduction

Helminthiases are among the most prevalent parasitic diseases globally, caused by nematodes, trematodes, and cestodes^1^. In Europe, infections with the nematode species *Enterobius vermicularis* (pinworm) predominate^2^ and generally do not pose a significant risk to the mother or unborn child^3^. In contrast, infections with some nematodes belonging to the group of soil-transmitted helminths (STH), such as hookworms, as well as *Schistosoma* species, have been associated with anemia and other pregnancy-related complications^4–7^.

Thus, safe and effective treatment and prophylaxis during pregnancy are of considerable clinical importance. In regions endemic for STH or schistosomiasis, the WHO therefore recommends mass drug administration programs, including pregnant women after the first trimester and under specific conditions^8,9^. In non-endemic regions such as Europe, treatment of affected pregnant women is indicated and assessed on a case-by-case basis. This also applies to cestode infections such as echinococcosis, which often similarly require treatment with anthelmintics during pregnancy, often over a prolonged period.

Depending on the underlying infection, various anthelmintic agents are available as therapeutic options, including albendazole, mebendazole, praziquantel, pyrantel and pyrvinium. Knowledge about the use of these drugs in pregnancy is often based on experience from countries with endemic STH-infections and schistosomiasis and on mass deworming programs. However, these studies predominantly address therapeutic efficacy, whereas data on safety, especially during the first trimester of pregnancy, remain limited.

The most evidence regarding use in pregnancy is available for the benzimidazole agent mebendazole. Although concerns about potential reproductive toxicity have been raised based on in vitro and animal data, human studies have not confirmed an elevated risk of adverse pregnancy outcomes^10–12^. Similar findings were reported for albendazole, another benzimidazole, although the dataset is more limited^13–16^.

Praziquantel has been used for over three decades, and available case studies, cohort studies, and randomized controlled trials consistently demonstrate no increased risk during pregnancy^17,18^. The safety of pyrvinium, primarily used for pinworm infection, has been examined in a Danish cohort study^11^. For pyrantel, safety data are based only on a small retrospective case series in the third trimester^19^ and limited experience from mass deworming programs in the second and third trimester.

Despite previous investigations, uncertainty concerning anthelmintic use during pregnancy remains, especially in the first trimester. The present study therefore aims to contribute to a better understanding of the safety profile of anthelmintic drugs during pregnancy, with a specific focus on the potential risks of major congenital malformations and spontaneous abortions associated with early gestational use.

## Methods

In this observational cohort study, pregnancies systemically exposed to anthelmintic drugs (albendazole, mebendazole, praziquantel, pyrantel and pyrvinium) were analyzed. The study was based on data collected by the Embryotox Center of Clinical Teratology and Drug Safety in Pregnancy (Embryotox) during the study period from January 1, 2000 to February 28, 2023. In addition, a structured literature search was conducted in MEDLINE to contextualize these findings within the available published data.

Embryotox is a national counselling center providing information on drug safety during pregnancy and lactation. Data obtained through its advisory service and subsequent pregnancy follow-up were recorded in the Embryotox database (VigilanceONE, PharmApp Solutions GmbH, Erkrath, Germany) and formed the basis of this study project. As our center primarily serves the German-speaking population, the majority of patients were expected to originate from Germany. In addition, Embryotox acts as a pharmacovigilance center, reporting any suspected adverse drug reactions to the respective federal higher authorities.

During the initial phone consultation and counselling with pregnant women or their health care professionals, physicians or pharmacists documented standard patient data, including contact information, maternal characteristics, estimated date of birth, gestational week (GW), current medication (dose, duration, indication), folic acid intake, and exposure to nicotine, alcohol, or illicit drugs, as well as obstetric history. If consent was given, standardized pregnancy follow-up was performed to document the course and outcome of pregnancy. Follow-up questionnaires, completed approximately eight weeks after the estimated date of birth, collected information on neonatal outcomes (date and GW at birth, biometric data, Apgar scores, umbilical cord pH, and congenital anomalies). Additional drug exposures during pregnancy were also recorded. If no response was received within four weeks, reminders were sent. Cases without feedback were considered as “lost to follow-up.” All data were checked for completeness and plausibility. Follow-up data were reviewed and processed by physicians, pharmacists, and medical documentation specialists; medical reports were requested in cases of irregular findings. This structured follow-up procedure ensured a high standard of data quality. A detailed description of Embryotox procedures can be found in an overview article^20^.

Study datasets were retrieved from the Embryotox database using predefined criteria. Only prospectively ascertained cases with adequate data and completed follow-up were included in the analysis. Since the study focused on safety aspects of anthelmintic drug exposure during pregnancy rather than the appropriateness of their indication, all cases with documented exposure to one of the five investigated agents were included, regardless of the underlying diagnosis. Retrospective reports, where the pregnancy outcome was already known at the time of notification, were analyzed separately due to the inherent reporting bias toward adverse outcomes.

The primary study endpoints were major congenital malformations and spontaneous abortion following first-trimester exposure to anthelmintic drugs. Reported congenital anomalies were classified as major or minor according to guidelines of the European network of population-based registries for the epidemiological surveillance of congenital anomalies (EUROCAT 1.5)^21^ by two independent Embryotox experts (pediatrics and human genetics) who were blinded to the exposure status; discrepancies were resolved by a third blinded expert. Chromosomal anomalies were classified as a separate category in accordance with the EUROCAT guideline and were analyzed separately from major structural birth defects. Secondary outcomes included birth weight (BW), head circumference, and preterm birth.

First-trimester exposure was defined as drug intake between GW 2 + 0 (presumed conception) and GW 12 + 6 after the first day of the last menstrual period. Spontaneous abortion was defined as pregnancy loss before GW 24 + 0 or of a fetus < 500 g. Stillbirth was defined as fetal death ≥ 500 g or ≥ 24 weeks’ gestation. Preterm birth was defined as delivery before 37 + 0 weeks.

Approval was obtained from the ethics committee of the Charité **–** Universitätsmedizin Berlin (EA2/069/23). The study was registered at the German Clinical Trial register (DRKS00032898, date of registration: 04.03.2024). The study was performed in accordance with the relevant guidelines and regulations. Informed consent was obtained from all participants included in the study.

For the evaluation of the prospective cohort, descriptive statistics were applied. The standard deviation scores (SDS) for birth weight and head circumference were calculated based on percentiles derived from the German perinatal survey^22,23^. Binomial 95% confidence intervals (CI) were calculated for birth defect rates using the exact method. Confidence intervals are presented without adjustment for multiplicity and should not be used to infer definitive statistical significance or for formal hypothesis testing. All statistical computations were performed in R version 4.3.2.^24^.

## Results

**Fig. 1:**
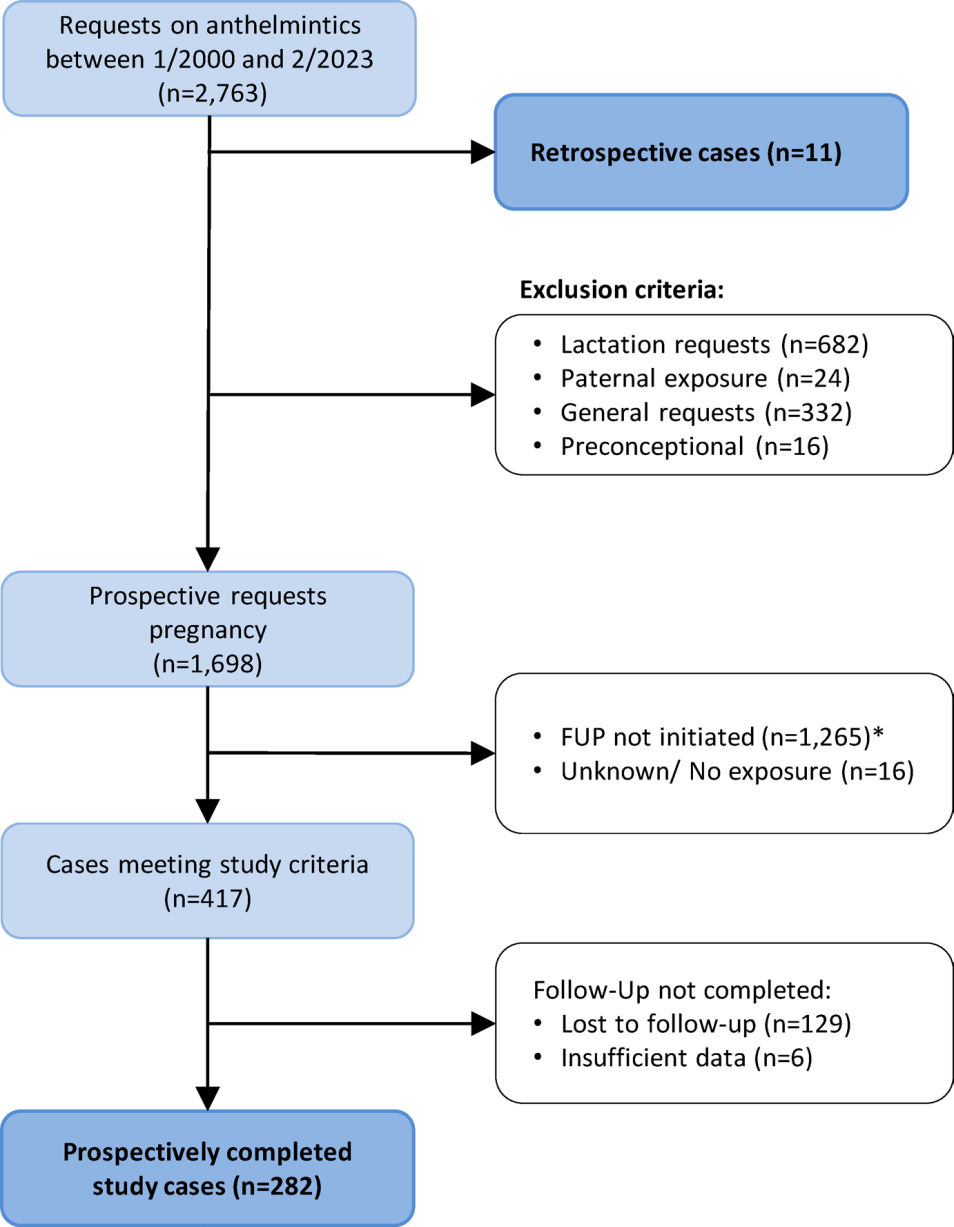
Flowchart explaining inclusion criteria for anthelmintic cohort. *n*, number of cases. *Of the 1,265 pregnancies for which prospective follow-up was not initiated, 93.5% had exposure during the 2nd/3rd trimester only.

In total, Embryotox received *n* = 2,763 requests concerning anthelmintics during the study period, thereof *n* = 1,698 prospectively. Out of these, *n* = 417 met the study criteria. The final cohort consisted of *n* = 282 cases, as *n* = 129 were considered lost and *n* = 6 cases had insufficient data (Flowchart Fig. 1). Details on exposure by active substance and trimester of intake are presented in Table 1.

**Table 1:** Number of exposed pregnancies categorized by anthelminthic drugs and trimester of exposure.

Study medication	Total exposed pregnancies^a^, *n*	1st trimester only, *n*	1st and 2nd/3rd trimester, *n*	2nd/3rd trimester only, *n*	Trimester unknown, *n*
Mebendazole	149	76	7	65	1
Pyrvinium	81	36	10	34	1
Pyrantel	24	18	1	5	0
Albendazole	12	8	2	2	0
Praziquantel	6	4	0	2	0
> 1 substance	10^b^	5	3	2	0
Total	282	147	23	110	2

*n* number of cases.

^a^Including five twin pregnancies: two twin pairs with 1st trimester mebendazole exposure, one pair with 1st trimester exposure to both mebendazole and pyrantel, one pair with pyrvinium exposure in the 2nd/3rd trimester, and one pair with mebendazole exposure in the 2nd/3rd trimester.

^b^Combination therapy: see Table S2 for details on the cases with exposure to more than one anthelmintic.

### Maternal characteristics

The median age of women was 33.5 years. 58.5% of the patients had a university degree. The vast majority of women did not consume nicotine (91.0%) or alcohol (95.3%) during pregnancy. A high proportion of pregnancies were planned (96.5%). These data were broadly consistent with the maternal characteristics of historical reference cohorts from previous Embryotox studies, e.g.^25^. Further information on maternal characteristics is given in Table S1.

### Treatment indications and exposure to anthelmintics

Most patients were treated due to infection with *Enterobius vermicularis* (*n* = 201). Of these, 100 patients were treated exclusively with mebendazole, 76 with pyrvinium, 19 with pyrantel and 2 with albendazole. Two patients received a combination of mebendazole and pyrvinium, and another two were treated with pyrvinium and pyrantel. The second most common infection was ascariasis (*n* = 26), with 21 patients treated with mebendazole, 2 with pyrvinium, and one each with albendazole and pyrantel, respectively. One patient received both mebendazole and pyrvinium. Four women diagnosed with echinococcosis were all treated with albendazole, one of them in combination with praziquantel. Three women had schistosomiasis (all treated with praziquantel) and another three were diagnosed with taeniasis (two treated with albendazole and one with praziquantel). One patient each was diagnosed with clonorchiasis (treated with albendazole and praziquantel), filariasis (treated with mebendazole), trichuriasis (treated with mebendazole), dibothriocephalosis (treated with mebendazole and pyrantel) and hookworm (treated with pyrantel) infection. One additional case involved albendazole use for a *Giardia lamblia* infection. Although *Giardia lamblia* is not a helminth, this case was retained in the analysis as the inclusion criteria required documented exposure to the study medication, irrespective of the underlying diagnosis. In the remaining pregnancies (*n* = 39), the underlying diagnosis was not specified or, in two cases, the exposure was reported to be inadvertent. Among these 39 cases, 37 patients received monotherapy: 26 received mebendazole, 3 pyrvinium, 3 albendazole, 3 pyrantel, and 2 praziquantel. The remaining two patients received combination therapy.

Full information on dose and duration of treatment was available for 147 monotherapy cases and is summarized in Table S2, Part A. Reported regimens varied considerably depending on the anthelmintic agent and underlying infection. The majority of patients (*n* = 168/233, 72.1%) received short-course treatment of one to three days. Ten patients received a combination of two anthelmintic agents (Table S2, Part B).

### Pregnancy outcome

Among the *n* = 282 included pregnancies, there were *n* = 262 live-born infants, including five sets of twins. After first-trimester exposure to the study medication *n* = 150 infants were live born. Twenty pregnancies (*n* = 20) ended in spontaneous abortions, 16 occurred in the first trimester and 4 in the second trimester, respectively. Further details on spontaneous abortions by anthelmintic agent and trimester of exposure are provided in Table 2. Of these four, two followed premature rupture of membranes in the 16th and 18th GW, respectively. In the third, cervical dilation was reported in the 18th GW and in the fourth, trisomy 18 was diagnosed in the fetus. In three of the study cases, there was an interval of at least 10 weeks between the intake of an anthelmintic and spontaneous abortion; therefore, a causal relationship appeared unlikely. In one case, pyrantel was taken approximately one week before spontaneous abortion.

**Table 2:** Pregnancy outcomes.

	Total exposed pregnancies (*n* = 282)^a^	1st trimester exposure (*n* = 170)	2nd/3rd trimester exposure only (*n* = 110)	Exposure in unknown trimester (*n* = 2)
SAB	20^b^	18	1	1
ETOP	4^c^	4	0	0
Stillbirths	1	1	0	0
Live births	257	147^d^	109	1
Thereof infants with major BD	10	8	2	0

*BD* birth defects, *ETOP* elective termination of pregnancy, *SAB* spontaneous abortions, *n* number of cases.

^a^Includes 23 cases with additional exposure after 1st trimester; 2 cases with exposure in unknown trimester.

^b^11 SAB after 1st trimester mebendazole exposure; four SAB after 1st trimester pyrvinium exposure; three SAB after 1st trimester pyrantel exposure, thereof one case with trisomy 18; one SAB after exposure to pyrvinium in unknown trimester; one SAB after pyrantel exposure in 2nd trimester.

^c^one ETOP after albendazole and praziquantel exposure in 1st trimester and three after 1st trimester mebendazole exposure; thereof one with trisomy 21.

^d^resulting in 150 live-born infants, of which 75 followed exposure to mebendazole, 47 to pyrvinium, 21 to pyrantel, 10 to albendazole and 4 to praziquantel; 7 of these infants were exposed to more than one anthelmintic.

Four patients underwent an ETOP (*n* = 4), three of which were performed in the first trimester for personal reasons. In another case, an ETOP was performed after a trisomy 21 diagnosis.

There was one stillbirth (*n* = 1) at GW 28. The overweight patient had a single dose of pyrvinium in GW 8 and additional co-medication for hypothyroidism (levothyroxine and potassium iodide daily).

### Neonatal characteristics

The neonatal characteristics of the study cohort are summarized in Table S3. Among the 262 live-born infants, 14 (5.3%) were born preterm (i.e., before completed 37 weeks of gestation). The average SDS of the children’s head circumference was 0.08 and the average SDS of the children’s birth weight was 0.25.

### Birth defects

A total of n = 8 major congenital malformations occurred in the 150 live-born infants (including three twin pairs) exposed to anthelmintics in the first trimester (5.33%; 95% CI 2.33**–**10.24). For a detailed case description, including documented co-medication, see Table 3.

**Table 3:** Description of major birth defects in the cohort exposed to anthelmintics.

Infant	Exposure (GW)	Substance (duration, dose)	Treatment indication	Pregnancy outcome, GW at birth, weight, sex	Major birth defects; additional minor anomalies	Co-exposure* in 1st trimester (periconceptional included)
1	3	Pyrantel (1 d, n/a)	Enterobiasis	Live born, GW 40, 50–75% perc., male	Major: Aplasia cutis of the scalp	Thiamazole (GW 0 to 40); Ibuprofen (GW 0 to 27 as needed)
2	6	Pyrantel (1 d, 750 mg/d)	Enterobiasis	Live born, GW 40, 25–50% perc., male	Major: Aplasia cutis of the scalp Minor: Small unilateral naevus on the forearm	Not reported
3	6	Mebendazole (6 d, n/a)	Enterobiasis	Live born, GW 30, 25–50% perc., male	Major: Hypospadias, Renal duplication	Not reported
4	6–8	Mebendazole (6 d, 100 mg/d)	Enterobiasis	Live born, GW 40, 75–90% perc., female	Major: AVSD	Fosfomycin (GW 8)
5	7	Mebendazole (1 d, 100 mg/d)	Enterobiasis	Live born, GW 40, 10–25% perc., female	Major: TGA	Ibuprofen (single dose in 1st trimester)
6	8	Mebendazole (1 d, 100 mg/d)	Enterobiasis	Live born, GW 40, 25–50% perc., male	Major: Dextrocardia	Not reported
7	10	Pyrvinium (1 d, n/a)	Enterobiasis	Live born, GW 40, 50–75% perc., female	Major: VSD Minor: enlarged renal pelvis	Not reported
8	10–14	Mebendazole (3 d, 100 mg/d)	Unspecified helminth infection	Live born, GW 40, 50–75% perc., male	Major: VSD	Nitrous Oxide (GW 7)

*GW* gestational week, *AVSD* atrioventricular septal defect, *perc.* Percentile, *VSD* ventricular septal defect, *TGA* transposition of the great arteries, *excluding routine supplementation, e.g. folic acid.

Out of the 75 live-born infants following first-trimester mebendazole exposure,* n* = 5 major congenital malformations were reported (6.67%; 95% CI 2.20–14.88). These included three heart defects: transposition of the great arteries (TGA), ventricular septal defect (VSD) and atrioventricular septal defect (AVSD). There was also one child with an isolated dextrocardia, a lateralization anomaly and urogenital malformations in another male infant (hypospadias in combination with renal duplication).

Out of 21 live-born infants with first-trimester pyrantel exposure, *n* = 2 were reported with a major birth defect (9.52%; 95% CI 1.17–30.38). In both children, aplasia cutis of the scalp was reported. In one of these study cases, the patient also took 10 mg of thiamazole throughout pregnancy due to Graves’ disease and used ibuprofen “on demand’’ until GW 27.

Among 47 live-born children exposed to pyrvinium in the first trimester, one major congenital malformation was reported (2.13%; 95% CI 0.05–11.29). The affected child showed a heart defect (VSD) along with pyelectasia. No birth defects were observed among 10 live-born children exposed to albendazole in the first trimester (95% CI 0–30.85) and 4 live-born children exposed to praziquantel in the first trimester (95% CI 0–60.24).

### Retrospective cases

In 5 of the 11 retrospectively reported pregnancies exposure to anthelmintics took place during the first trimester. Out of these, three spontaneous abortions between GW 8 and 11 were reported after exposure to pyrvinium, mebendazole and pyrantel respectively. Two live-born infants were exposed to pyrvinium during the first trimester. In one infant, a congenital hearing impairment was diagnosed, while the second showed no adverse findings. Relevant co-medication was not reported.

## Discussion

We evaluated a cohort of pregnancies exposed to five different anthelmintic agents, thereby contributing to the available experience on the use of anthelmintics during pregnancy. Before discussing the findings, it should be noted that all observations in this cohort are descriptive in nature. Given the small subgroup sizes and the absence of a comparator group, no conclusions regarding the teratogenic risk of the investigated drugs can be drawn.

### Major congenital anomalies and pregnancy outcomes

Among anthelmintic drugs, mebendazole has the most experience regarding use during pregnancy. Study data on first-trimester outcomes are available from two cohort studies conducted in Israel and Denmark^10,11^, a cross-sectional study from Sri Lanka^26^, and a Hungarian population-based case–control study^27^, comprising a total of over 1,500 mebendazole-exposed pregnancies. None of these studies demonstrated an increased risk of congenital malformations. There is more extensive experience with mebendazole use after the first trimester, including two randomized controlled trials conducted in endemic settings^28,29^. These studies did not suggest an increased risk of adverse pregnancy outcomes; however, the primary focus was on the therapeutic efficacy and potential benefits of treatment. A comprehensive systematic review further concluded that current data provide no evidence of increased risks from mebendazole use during pregnancy^12^. From a pharmacokinetic perspective, mebendazole exhibits limited oral bioavailability (< 10%) and undergoes extensive first-pass metabolism, resulting in only low maternal plasma concentrations after standard treatment doses^30^.

Among 75 live-born infants following first-trimester mebendazole exposure in the Embryotox cohort, five major congenital malformations were observed (6.67%; 95% CI 2.20–14.88). Three of these were congenital heart defects (a VSD, an AVSD, and a TGA). In addition, isolated dextrocardia, a lateralization anomaly, was present in another infant. One further child was affected by hypospadias and a renal duplication.

Congenital heart defects represent the most common category of major birth defects in the general population, accounting for over 30% of all major birth defects^31–33^, and their occurrence in this cohort is therefore not unexpected. However, given the small sample size and wide confidence interval (95% CI 2.20–14.88), no firm conclusions regarding a specific teratogenic risk can be drawn. To our knowledge, neither an increased overall risk of major congenital malformations nor a specific increased risk for cardiac defects after first-trimester mebendazole use has been reported in the literature to date.

Albendazole is poorly absorbed (< 5%); however, it is rapidly and almost completely converted to its active metabolite, which reaches the systemic circulation in therapeutically relevant concentrations^34,35^. Nevertheless, none of the studies available to date have suggested an increased risk of adverse pregnancy outcomes: approximately 200 pregnant women received treatment with albendazole during the first trimester, as reported in data from a Korean cohort study^15^, a Ghanaian cohort study^13^, and a case series from the UK^14^. As with mebendazole, experience with albendazole beyond the first trimester is more extensive due to its use in mass deworming programs. Findings from various randomized controlled trials^36–40^ and cohort studies^41,42^, investigating the efficacy and safety of albendazole in the second and third trimesters, similarly found no evidence of fetal risk. No particular risks were reported by two comprehensive systematic reviews^12,16^. In the Embryotox cohort, no major birth defects were observed in 10 live-born infants exposed to albendazole in the first trimester. This included one case of albendazole exposure throughout the entire pregnancy due to echinococcosis.

Existing data on the use of pyrvinium to treat *Enterobius vermicularis* infection during pregnancy is primarily based on a Danish cohort study^11^. In this study, involving *n* = 1,588 pregnancies, of which *n* = 445 were exposed during the first trimester, no increased risk of congenital malformations or stillbirths was observed. The observations from the Embryotox cohort are consistent with these findings. One major birth defect was reported among 47 infants exposed to pyrvinium in the first trimester, namely a VSD, accompanied by pyelectasia, the latter considered a minor anomaly. It should also be noted that pyrvinium is administered as the pamoate salt, which is essentially not absorbed from the gastrointestinal tract following oral administration^43^.

In contrast to the other anthelmintic agents discussed here, praziquantel is well absorbed orally (> 80%), although pronounced first-pass metabolism considerably reduces systemic exposure^44^. So far, in three randomized controlled trials conducted in the Philippines, Gabon, and Uganda (Mother and Baby Study)^45–47^, two cohort studies from Saudi Arabia^48,49^, case reports and case series, and reassuring animal data^50–54^, no signals have emerged to suggest risks associated with praziquantel intake during pregnancy^17,18^. Nevertheless, data on first-trimester exposure remain limited, with only approximately 50 cases reported to date. In the Embryotox study cohort, all four live-born infants with first-trimester praziquantel exposure were born without major congenital malformations.

To our knowledge, the only published clinical data on pyrantel exposure during pregnancy come from a retrospective case series describing 15 pregnant women with biliary ascariasis who received a single oral dose of pyrantel during the third trimester. The authors reported one preterm delivery at 34 weeks of gestation, which they attributed to the underlying disease rather than to pyrantel treatment^19^. Regarding pharmacokinetics, pyrantel pamoate undergoes only minimal gastrointestinal absorption, resulting in a low maternal systemic exposure^55^. Within the Embryotox cohort, two of 21 live-born infants exposed to pyrantel in the first trimester were affected by aplasia cutis, a rare congenital birth defect with an estimated prevalence of 1**–**5 per 10,000 live-births^56,57^.

The pathophysiology of aplasia cutis congenita is incompletely understood and considered multifactorial. Proposed contributors include genetic factors, intrauterine infections, and teratogen exposure^56^. For aplasia cutis of the scalp, one discussed hypothesis implicates mechanical tension during the period of rapid cranial growth, approximately between the 10th and 15th GW^56,58^. In the two cases reported here, pyrantel exposure occurred in the 3rd and 6th GW, respectively. From an embryogenetic perspective, this exposure seems to be too early to explain the phenotype. The occurrence of aplasia cutis in one infant may be explained by the mother’s additional intake of thiamazole throughout the entire pregnancy, given that an association between thiamazole and an increased risk of aplasia cutis following first-trimester exposure is known to exist, however, the pathomechanism remains unclear to date^59^. In contrast, no co-exposure was identified in the second case of aplasia cutis. This might suggest a potential signal but could also be mere coincidence.

The expected baseline risk for spontaneous abortion ranges from 15**–**20%^60,61^. In the present cohort, a total of 20 spontaneous abortions were reported across all five anthelmintic subgroups (*n* = 20/282, Table 2). Since the median gestational age at first contact was 10 weeks, and it is known that the majority of spontaneous abortions occur in early stages in pregnancy, early losses are likely underrepresented^62^. Consequently, the observed rate cannot be interpreted as reflecting the true risk of pregnancy loss, and a drug-specific evaluation is not possible.

Published data on pregnancy loss following anthelmintic medication are limited and insufficient to characterize the risk for each agent individually. In general, previously published data do not suggest an increased risk of spontaneous abortion to our knowledge^13,15,16,38^. However, one case series describes course and outcome of pregnancies in a very specific cohort of 27 patients diagnosed with cystic echinococcosis^63^. Five patients received albendazole, four of whom were in the first trimester. These five pregnancies resulted in spontaneous abortion. Whether this reflects a drug effect or whether the negative pregnancy outcome was influenced by the underlying condition itself remains uncertain. In the Embryotox study cohort, there was no pregnancy loss among patients exposed to albendazole for different indications.

A review of dosing regimens in all study cases with major congenital malformations and spontaneous abortions (Table S2) showed no substantial deviation from the cohort median, with the exception of one late pregnancy loss following extended mebendazole treatment (600 mg/d for 13 days between GW 5 and 7) for ascariasis in a patient with uterine leiomyoma^64^. In this case, the clinical course (premature cervical dilation in GW 18) argues against a causal relationship to mebendazole exposure.

In the Embryotox study cohort, one stillbirth occurred at 28 weeks of gestation. The patient had received a single dose of pyrvinium in the first trimester and was categorized as overweight with a BMI of 28, a known risk factor for pregnancy complications^65^. Therefore, causal association with first-trimester intake is considered unlikely.

The observed proportion of preterm births in the present cohort (5.3%) was below the background rate of 6.0–6.8% reported for the general German population in recent years^66^. Although a substance-specific evaluation was not feasible given the small subgroup sizes, the overall data do not indicate an elevated risk of preterm delivery following anthelmintic exposure during pregnancy.

As reported in the results section, retrospective case reports of anthelmintic exposure in the first trimester were evaluated separately, as they can be useful for signal detection. Of the five retrospectively reported cases, three pregnancies ended in a spontaneous abortion and one infant had a congenital hearing impairment, not observed in the prospective study cohort. These data do not provide additional information on possible signals.

### Strengths and limitations

As previously described in detail, general limitations inherent to observational cohort studies conducted by Teratology Information Services, such as selection bias or response bias, must be considered^67^. Furthermore, due to the limited sizes of the exposed sub-cohorts, any risk calculations would lack sufficient statistical power, and comparisons with control groups would not be statistically valid. For illustration, detection of a fivefold increase in the overall rate of major birth defects would require approximately 85 exposed pregnancies per group, assuming a baseline prevalence of 3% (α = 0.05, 80% statistical power, two-sample comparison). Even our largest subgroup, 75 live-born infants exposed to mebendazole in the first trimester, is therefore not suitable to detect such an increase. Detection of specific birth defects, such as cardiac anomalies with a baseline prevalence of ~ 1%, would require substantially larger cohorts. Consequently, our analysis was limited to descriptive evaluation and detection of risk signals rather than quantitative risk assessment. The confined case number and the absence of a comparator group are key limitations of this study and limit the interpretability of the observed rates. Where comparisons with the general population prevalence rates are referenced, these are provided solely for contextual orientation. We are aware that the Embryotox cohort is not comparable to the general population in all characteristics, as e.g. the level of education is known to be higher^68^. Reported co-medication and other factors (such as genetic predisposition) may additionally influence the pregnancy outcome. Furthermore, confounding by indication cannot be excluded, due to heterogeneity of underlying parasitic infections and their respective treatment protocols. It is challenging to disentangle these different aspects from the underlying drug effect.

Despite these limitations, the Embryotox study offers several notable strengths that set it apart from previously published data. Data were prospectively collected according to a standardized internal protocol to ensure consistency and high quality. Detailed medication histories were obtained, including dosing regimens, concomitant therapies, maternal characteristics and relevant potential risk factors. All congenital anomalies were classified in a blinded manner by two independent experts from our institute, minimizing the likelihood of misclassification bias.

## Conclusion

The present analysis provides an overview of pregnancy outcomes following first-trimester exposure to five different anthelmintic agents. Anthelmintics are a heterogeneous group of drugs with differing mechanisms of action, chemical structure, and available data on use in pregnancy. Each substance requires an individual risk–benefit assessment. The major congenital anomalies observed with mebendazole and pyrantel should be interpreted cautiously, given the limitations discussed. Further studies focusing on birth defects and pregnancy outcomes in larger cohorts are therefore needed.

## Supplementary Information

Below is the link to the electronic supplementary material.

Supplementary Material 1

## Data Availability

The data that support the findings of this study are available upon reasonable request from the corresponding author. The data are not publicly available due to privacy or ethical restrictions.
