# Supplementary material for: Anthelmintic medication with focus on first-trimester exposure: an evaluation of pregnancy outcomes based on the Embryotox cohort
Source: Sci Rep. 2026 Jun 14;16:18373. doi: 10.1038/s41598-026-57350-3 (PMC13265764; doi:10.1038/s41598-026-57350-3)
Supplement: Supplementary file 1 — Supplementary Material 1 [file 41598_2026_57350_MOESM1_ESM.pdf]

Supporting Information Scientific Reports

Anthelmintic medication with focus on first-trimester exposure: an evaluation of pregnancy outcomes based on the Embryotox cohort

Laura Heineke[1], Evelin Beck[1], Reinhold Kreutz [2], Katarina Dathe*[1]

[1]Charité – University Medical Center Berlin, corporate member of Freie Universität Berlin and Humboldt-Universität zu Berlin, Institute of Clinical Pharmacology and Toxicology, Embryotox Center of Clinical Teratology and Drug Safety in Pregnancy, Augustenburger Platz 1, 13353 Berlin, Germany

[2]Charité – University Medical Center Berlin, corporate member of Freie Universität Berlin and Humboldt-Universität zu Berlin, Institute of Clinical Pharmacology and Toxicology, Charitéplatz 1, 10117 Berlin, Germany

Table S1: Maternal characteristics

	**Anthelmintics n, 282**
**GW at first contact, n**	282
Median (IQR)	10 (7-17.6)
**Age (years), n**	280
Median (IQR), (min-max)	33.5 (30-37) (16-44)
**Pre-pregnancy BMI, n**	246
Median, (kg/m²), (IQR)	22 (20.4-24.7)
**Educational level, n**	176
No leaving exam, n (%)	2 (1.1)
9 years exam, n (%)	5 (2.8)
10/11 years exam, n (%)	32 (18.2)
Secondary school exam, n (%)	34 (19.3)
Academic study, n (%)	103 (58.5)
**Smoking, n**	278
No, n (%)	253 (91)
≤ 5 cig/day, n (%)	9 (3.2)
> 5 cig/day, n (%)	16 (5.8)
**Alcohol, n**	278
No, n (%)	265 (95.3)
≤ 1 drink/day, n (%)	8 (2.9)
> 1 drink/day, n (%)	5 (1.8)
**Pregnancy wanted, n**	230
Yes, n (%)	222 (96.5)
Indifferent, n (%)	6 (2.6)
No, n (%)	2 (0.9)
**Previous pregnancies, n**	277
0, n (%)	62 (22.4)
1, n (%)	65 (23.5)
2, n (%)	75 (27.1)
≥ 3, n (%)	75 (27.1)

**Previous parities, n**	277
0, n (%)	72 (26)
1, n (%)	79 (28.5)
2, n (%)	80 (28.9)
≥ 3, n (%)	46 (16.6)
**Previous SAB, n**	277
0, n (%)	212 (76.5)
1, n (%)	42 (15.2)
≥ 2, n (%)	23 (8.3)
**Previous children with birth defect, n**	277
0, n (%)	270 (97.5)
1, n (%)	6 (2.2)
≥ 2, n (%)	1 (0.4)

Legend. The absolute number of evaluable parameters differ due to missing or imprecise values. BMI, body mass index; GW, gestational week; IQR, interquartile range; n, number of cases with available information; SAB, spontaneous abortions.

Table S2: Treatment details

**Part A – Monotherapy (n=147 cases with complete dose and duration data)**				
**Drug**	**Duration known (n=233)**	**Median duration, d (IQR)**	**Dose known (n=156)**	**Median dose, mg/d (IQR)**
Mebendazole	124	3 (3-6)	99	100 (100-200)
Pyrvinium	68	1 (1-2)	31	400 (350-450)
Pyrantel	24	1 (1-1)	13	750 (500-750)
Albendazole	11	3 (1-18.5)	10	400 (400-700)
Praziquantel	6	1 (1-1)	3	2400 (2100-3900)

**Part B – Combination Therapy (n=10 cases)**				
**Case**	**Drug 1 (trimester of exposure)**	**Duration drug 1, d**	**Drug 2 (trimester of exposure)**	**Duration drug 2, d**
1	Albendazole (2nd/3rd)	5	Praziquantel (2nd/3rd)	1
2	Pyrvinium (1st-3rd)	4	Mebendazole (3rd)	3
3	Pyrvinium (1st)	1	Pyrantel (1st)	1
4	Mebendazole (1st-3rd)	6	Pyrvinium (1st)	1
5	Mebendazole (2nd/3rd)	3	Pyrvinium (2nd/3rd)	1
6	Albendazole (1st)	34	Praziquantel (1st)	18
7 (twins)	Mebendazole (1st)	3	Pyrantel (1st)	n/a
8	Pyrvinium (1st)	1	Pyrantel (1st)	1
9	Pyrvinium (1st-3rd)	3	Pyrantel (1st)	1
10	Mebendazole (1st)	n/a	Pyrvinium (1st)	1

Legend. IQR, interquartile range; n/a, not available; Duration and dose statistics (Part A) are reported only for monotherapy cases with complete data. Median and IQR for praziquantel dose should be interpreted with caution (n=3). IQR 1-1 for praziquantel and pyrantel duration reflects that the majority of cases received single-day treatment. Part B cases are sorted by completeness of available data.

Table S3: Neonatal outcomes (including five pairs of twins)

	Anthelmintics n, 262
**GW at birth, n**	262
GW, median (IQR)	40 (39-40.4)
**Preterm birth, n**	262
Preterm (<GW 37), n (%)	14 (5.3)
At term, n (%)	248 (94.7)
**Child's sex, n**	262
Female, n (%)	123 (46.9)
Male, n (%)	139 (53.1)
**Child’s weight (g), n**	262
Median, (IQR)	3,500 (3,150-3,860)
**Child‘s length (cm), n**	261
Median, (IQR)	52 (50-53)
**Child’s head circumference (cm), n**	248
Median, (IQR)	35 (34-36)

Legend. GW, gestational week; IQR, interquartile range; n, number of neonates with available information
